# Diabetic Retinopathy Screening Ratio Is Improved When Using a Digital, Nonmydriatic Fundus Camera Onsite in a Diabetes Outpatient Clinic

**DOI:** 10.1155/2016/4101890

**Published:** 2016-01-21

**Authors:** Pia Roser, Hannes Kalscheuer, Jan B. Groener, Daniel Lehnhoff, Roman Klein, Gerd U. Auffarth, Peter P. Nawroth, Florian Schuett, Gottfried Rudofsky

**Affiliations:** ^1^Department of Medicine I and Clinical Chemistry, University Hospital Heidelberg, Im Neuenheimer Feld 410, 69120 Heidelberg, Germany; ^2^Department of Endocrinology and Diabetology, University Medical Center Hamburg-Eppendorf, Martinistraße 52, 20246 Hamburg, Germany; ^3^Department of Ophthalmology, University Hospital Heidelberg, Im Neuenheimer Feld 400, 69120 Heidelberg, Germany; ^4^Department of Medicine, Kantonsspital Olten, Baslerstrasse 150, 4600 Olten, Switzerland

## Abstract

*Objective*. To evaluate the effect of onsite screening with a nonmydriatic, digital fundus camera for diabetic retinopathy (DR) at a diabetes outpatient clinic. *Research Design and Methods*. This cross-sectional study included 502 patients, 112 with type 1 and 390 with type 2 diabetes. Patients attended screenings for microvascular complications, including diabetic nephropathy (DN), diabetic polyneuropathy (DP), and DR. Single-field retinal imaging with a digital, nonmydriatic fundus camera was used to assess DR. Prevalence and incidence of microvascular complications were analyzed and the ratio of newly diagnosed to preexisting complications for all entities was calculated in order to differentiate natural progress from missed DRs. *Results*. For both types of diabetes, prevalence of DR was 25.0% (*n* = 126) and incidence 6.4% (*n* = 32) (T1DM versus T2DM: prevalence: 35.7% versus 22.1%, incidence 5.4% versus 6.7%). 25.4% of all DRs were newly diagnosed. Furthermore, the ratio of newly diagnosed to preexisting DR was higher than those for DN (*p* = 0.12) and DP (*p* = 0.03) representing at least 13 patients with missed DR. *Conclusions*. The results indicate that implementing nonmydriatic, digital fundus imaging in a diabetes outpatient clinic can contribute to improved early diagnosis of diabetic retinopathy.

## 1. Introduction

About 382 million people suffer from diabetes. Over the last decades, it has become one of the most common diseases not only in the western world [1]. Diabetes comes along with an increase of diabetes complications, which lead to a loss of quality of life and finally to premature mortality [2–4].

Therefore, regular screening intervals for diabetic complications are essential. Screening on a regular, usually yearly basis for diabetic neuro- and nephropathy is mostly performed by the general practitioner or diabetologist [5, 6]. Concerning diabetic changes of the ocular fundus, patients are often referred to an ophthalmologist, which requires a good network for exchange of relevant findings as well as possible arising consequences for adaption of diabetes therapy and control of other risk factors [7].

The International Diabetes Federation (IDF) guidelines recommend annual screenings for diabetic retinopathy (DR) and screening within 3 to 6 months in case of a deterioration of the eye fundus since the last examination [8]. Screening for DR aims to detect sight threatening lesions which can effectively be treated [9]. However, some patients do not regularly attend screening intervals [10]. Persons who missed one or more years of retinopathy screening represent a high-risk group with a greater probability of having developed retinopathy in the meantime [11]. The barriers to receive regular eye examinations are multifactorial and vary in different studies [10–14]. Lack of awareness of the effects of DR on visual acuity, the absence of an ophtalmologist, no possibility of transportation, long waiting time, and physical disability are some commonly reported barriers for not receiving regular eye care [14, 15]. Since the increasing number of patients with diabetes worldwide will exceed ophthalmologists' capacities, this problem is likely to worsen in the following years [16, 17]. Another problem might arise from earlier manifestation of the disease. Diabetes is diagnosed more and more in people that are younger and still involved in working life processes with chronic lack of time, lack of long-term planning, and resistance to lifestyle changes [18]. Therefore, other screening requirements, for example, screening for DR through the general practitioner or the diabetologist, might improve patient care [19].

In this study, we analyzed whether screening for DR in a specialized diabetes center might improve early diagnosis of DR. Therefore, a nonmydriatic fundus camera was implemented in the diabetes outpatient clinic at the University Hospital of Heidelberg. Nonmydriatic stereoscopic retinal imaging has been demonstrated to be a reliable, cost-effective, highly sensitive, and specific method for DR [20–22], which can be carried out safely by adequately trained nonophthalmologists [23–26]. Further nonmydriatic screening comes along with a higher level of comfort and is less time consuming, with a photography session taking no longer than 2 minutes, compared to mydriatic fundus screening, for which pupillary dilation alone takes additional 20 to 30 minutes [27]. Furthermore, after pupillary dilation patients are also decreased in visual function for several hours and consequently limited in means of transport [28].

## 2. Research Design and Methods

A total of 502 patients with type 1 or type 2 diabetes were included in this study over a time period of six months. Patients were recruited from the outpatient clinic of the Department of Endocrinology at the University Hospital of Heidelberg, Germany. Eligibility criteria included patients with established diabetes mellitus type 1 or type 2, age of 18 years or older, and the ability to cooperate. Monocular vision was an exclusion criterion. The following data were collected from each patient: age, duration of diabetes, type of treatment, associated systemic risk factors, and history of eye treatment or examinations in the past. Data of all screened patients were documented in an ad hoc generated database. This database was built up of utilizable evaluation scores, and preexisting medical reports from internal and external sources were used as source data. The study protocol was approved by the ethics committee of the University of Heidelberg. Informed consent was given by all study participants.

### 2.1. Funduscopy

All 502 patients underwent single-field 45-degree (in smaller pupil diameter mode: 37°) retinal photography with a nonmydriatic auto fundus camera (Nidek/Oculus AFC-230/210, NIDEK Co., Ltd., Japan) linked to a high resolution digital SLR (single lens reflex) camera (21.8 megapixel full frame sensor, Canon EOS 5D Mark II, Canon Deutschland GmbH, Krefeld, Germany). The images were captured with central focus on the macula including the optic disk. They were stored and sorted through an incorporated data filing system, called NAVIS-Lite. Photography was performed without pupillary dilation. To allow for stable quality, all images were taken by the same trained technician throughout the study. Before commencing the study, the medical technician was instructed in using the camera and interpreting the retinal photographs, until he felt safe on carrying out the examination. Studies verify that this way of screening correlates with a high level of accuracy [24, 25]. If the original image was judged unsatisfactory by the photographer, the image acquisition process was repeated. The maximum number of attempts in order to achieve a satisfactory picture was set to three. In total the examination process did not exceed five minutes of time. The trained technician and an ophthalmologist evaluated the fundus photographs separately by using a self-developed questionnaire as published previously [25].

### 2.2. Diagnostic Criteria

**Figure 1:**
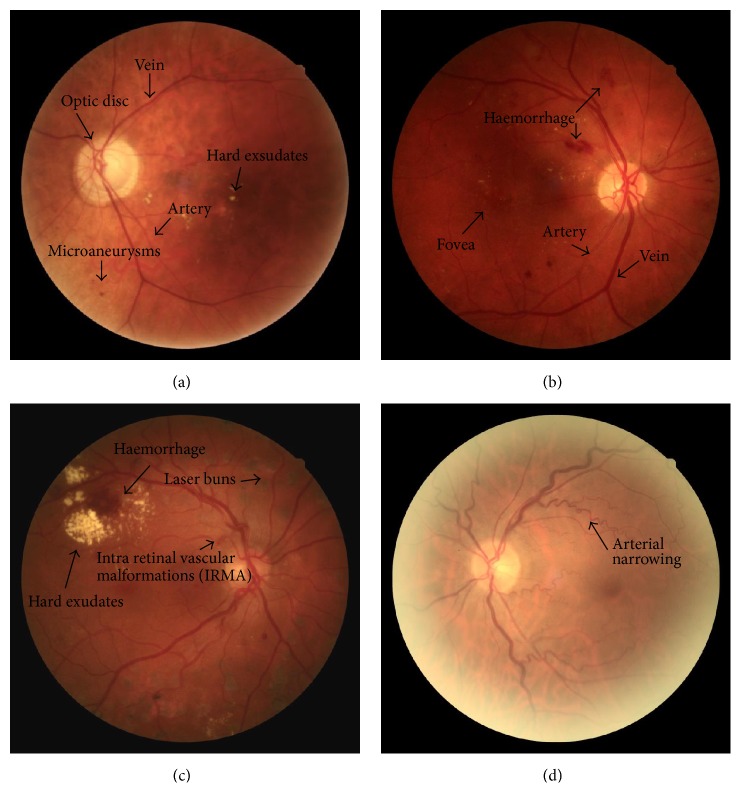
Four different pathologies which can be detected in nonmydriatic funduscopy. (a) Mild nonproliferative retinopathy with hypertensive fundus. (b) Moderate nonproliferative retinopathy. (c) Proliferative retinopathy after laser photocoagulation. (d) Hypertensive retinopathy grade I.

Diabetic retinopathy was defined according to the International Clinical Diabetic Retinopathy Disease Severity Scale [29]. The severity of diabetic retinopathy was assessed after taking at least one meaningful retinal image of each eye with a nonmydriatic fundus camera. The presence or absence of diabetic retinopathy was determined at the end of every screening day. Feedback on images and validation of the diagnosis DRs was performed once weekly by an ophthalmologist with special interest in diabetic retinal disease. Depending on the results, patients were differentiated into the two groups “no retinopathy” and “retinopathy.” Within the “retinopathy” group, two categories “preexisting retinopathy” and “new retinopathy” were established. Patients were then stratified according to the severity of retinopathy into mild, moderate, and severe “nonproliferative retinopathy” (Figures 1(a) and 1(b)), as well as into “proliferative diabetic retinopathy” and “hypertensive retinopathy” (Figures 1(c) and 1(d)).

All patients were screened at the department within the last 12 months for diabetic neuro- or nephropathy. The latest results were taken for the evaluation of this study. Diabetic nephropathy was defined as microalbuminuria of more than 20 mg/L in two of three samples of morning urine obtained within twelve consecutive months [30]. Patients with urinary infections were excluded. Laboratory albumin concentration in spot urine was measured at the central laboratory of the University Hospital of Heidelberg.

Absence or presence of neuropathy was assessed using neuropathy symptom score (NSS) and neuropathy disability score (NDS) [31]. Hypertension was defined as present if systolic blood pressure was >140 mmHg and diastolic blood pressure was >90 mmHg [32] or if the participant was taking antihypertensive drugs. Blood pressure was measured in a sitting position from the left arm, using an electronic blood pressure cuff. BMI was calculated as weight (kg)/height (m)². Patients' weight and height were measured by the same trained technician. Plasma glucose, fasting serum total cholesterol, and LDL and HDL cholesterol, as well as triglycerides, urinary albumin, and creatinine were determined by enzymatic methods by the clinical laboratory of the University of Heidelberg, which is fully accredited.

### 2.3. Statistical Analysis

Statistical analysis was performed using specific, predefined parameters. We used descriptive analyses for characterizing the study population. Comparisons of categorical baseline characteristics between the patients with DR and without DR were conducted by chi-square test analysis. Continuous baseline variables were compared using *t*-test. All potential risk factors were analyzed as either binary (e.g., nicotine abuse yes/no) or linear traits for continuous variables (e.g., “age”).

Logistic regression models were used to estimate odds ratios (ORs) and 95% confidence intervals (CIs) for mean last eye screening within 12 months or longer than one year.

The normally distributed data for descriptive analysis was declared as mean ± standard deviation, unless stated otherwise. The percentage values have been rounded off to whole numbers. Since microvascular complications differ with respect to prevalence and incidence rates, the ratio of new to preexisting complications was built. It was assumed that the rates for DN and DP represent more or less the rate of natural progress, since both complications are screened on a regular basis in the study center.

Statistical significance level was considered at a two-side probability level of 0.05 or less. Statistical analyses were performed using Excel 2003 and SPSS (PASW Statistics 18, IBM Deutschland GmbH, Ehningen, Germany).

## 3. Results

The study was conducted as a cross-sectional, nonrandomized, noncontrolled, prospective study. Patients were recruited over a time period of six months.

Overall, 502 nonrelated Caucasian patients with type 1 (*n* = 112, 22.0%) and type 2 diabetes (*n* = 390, 78.0%) were included. The characteristics are shown in Table 1.

### 3.1. Prevalence and Incidence of Diabetic Retinopathy

When all patients with diabetes were analyzed, prevalence of DR was 25.0% (*n* = 126). DR was present in 35.7% (*n* = 40) of type 1 diabetics and in 22.1% (*n* = 86) of participants with type 2 diabetes.

The incidence rate for both types of diabetes was 6.4% (*n* = 32) with similar results for patients with type 1 (5.4%, *n* = 6) and type 2 diabetes (6.7%, *n* = 26). Therefore, 25.4% (*n* = 32) of all 126 DRs were newly diagnosed and unknown before. In detail, of the 6 patients with type 1 diabetes and new DR, all (100.0%, *n* = 6) had mild nonproliferative DR NPDR. There was no case of new proliferative DR PDR.

Of the 26 patients with type 2 diabetes and new DR, 96.1% (*n* = 25) had nonproliferative diabetic retinopathy (NPDR), of which 84.6% (*n* = 22) had a mild form of NPDR and 11.5% (*n* = 3) had a moderate form of NPDR. Furthermore, one patient (3.8%) with type 2 diabetes had proliferative diabetic retinopathy (PDR).

Additionally, signs for associated hypertensive retinopathy were found in 43.4% (*n* = 218) of all patients being present in 22.3% (*n* = 25) of the patients with type 1 diabetes and 49.5% (*n* = 193) of the patients with type 2 diabetes. In both types of diabetes the presence of hypertensive retinopathy correlated with blood pressure and the taking of blood pressure lowering medications (*p* ≤ 0.001).

Next, patients with newly detected DR were characterized: Patients with type 1 diabetes and new onset DR had significantly higher systolic and diastolic blood pressure, positive history of cerebrovascular disease, and significantly higher total and LDL cholesterol levels and were more likely to have microalbuminuria (Table 2) in comparison to patients without DR. Patients with type 2 diabetes and new onset of DR had a significant longer duration of diabetes, positive history of preexisting neuropathy, diabetic foot syndrome, and significantly higher hemoglobin A_1c_ levels (Table 3).

### 3.2. Prevalence and Incidence of Diabetic Nephropathy

Prevalence of nephropathy was 32.5% (*n* = 163) in all diabetics. In patients with type 1 diabetes 17.9% (*n* = 20) were affected, whereas 36.7% (*n* = 143) of patients with type 2 diabetes had DN.

In total, 17.8% (*n* = 29) of all nephropathies (*n* = 163) were newly diagnosed revealing an incidence rate of 3.6% (*n* = 4) in participants with type 1 diabetes and 6.4% (*n* = 25) in participants with type 2 diabetes.

### 3.3. Prevalence and Incidence of Diabetic Polyneuropathy (DP)

The prevalence of any DP in both types of diabetes combined was 63.5% with 37.5% (*n* = 42) in participants with type 1 and 71.0% (*n* = 277) in participants with type 2 diabetes. 16.6% (*n* = 53) of all neuropathies (*n* = 319) were newly diagnosed. The incidence rate was 2.7% (*n* = 3) in participants with type 1 diabetes and 12.8% (*n* = 50) in participants with type 2 diabetes.

As described above, 32 DRs were newly detected by using a nonmydriatic fundus camera with onsite screening. Since prevalence of all three microvascular complications is different due to underlying pathogenesis, progression rates were calculated (Table 4). However, since the time point of the last evaluation of the complications was not standardized and not comparable in the analysed patients, progression rates used here do not refer to a distinct time period but to new onset since the last examination. While progression rates for DN and DP were 21.6% and 19.9%, respectively, rate was higher for DR (DN versus DR: 21.6% versus 34.0%; *p* = 0.12; DP versus DR: 19.9% versus 34.0%; *p* = 0.03), suggesting that more DRs were detected than expected due to progress of disease.

### 3.4. Factors Contributing to Newly Detected Diabetic Retinopathy

Finally factors contributing to the diagnosis of new DR were analyzed. Since the number of patients with type 1 diabetes was rather small and characteristics for patients with type 1 and type 2 diabetes were different, only patients with type 2 diabetes were included for following analysis. Interestingly, younger age was found to be a risk factor for nonattendance of regular retinopathy screening intervals (*p* = 0.01, Table 5). However, the time period since last eye screening was only significantly longer in type 2 diabetics with newly described DR compared to those with preexistent DR (Tables 2 and 3), although the proportion of patients with no yearly screening was significantly higher in patients with newly diagnosed DR (established DR versus new diagnosed DR: 4.3% versus 15.6%; *p* = 0.03). Overall, 67 (13.3%) participants did not attend eye screening within the last 12 months. While younger age and shorter diabetes duration were associated with a higher probability for missing regular screening visits, gender had no influence (Table 5). When comparing patients at the age of 50 years or younger with patients older than 50 years, the younger age group had a higher proportion with missed yearly screenings (age ≤ 50 years versus age > 50 years: 28.9% versus 13.6%; *p* = 0.008). Since preexisting DR could have influenced attendance rates especially in the older age group, rates of missed yearly eye screenings with and without preexisting retinopathy were analyzed. However, the results remain unchanged (age ≤ 50 years versus age > 50 years: 32.6% versus 16.9%; *p* = 0.02).

## 4. Discussion

The results of the study show that onsite screening for DR with a nonmydriatic, digital fundus camera can contribute to early diagnosis of diabetic retinopathy in a diabetes outpatient clinic.

In 32 of the analyzed patients, diabetic changes of the retina were newly described, which represents a proportion of 25% of all retinopathies detected in this study. One part of those can be seen as natural progress since the last screening visit. Nevertheless, the other proportion represents so far missed complications, which have been detected due to the changed screening process with onsite fundus screening. In order to separate these two proportions, progression rates for DR, DN, and DP were calculated. Since DN and DP were screened on a regular basis at the study center, one can assume that all newly detected DNs and DPs represent the natural progress of the disease. Both progression rates were close to 20%. Therefore, also a natural progression rate of 20% for DR was assumed. Since 94 DRs were preexistent before the study one would expect 19 newly detected DRs due to the natural progress. Since 32 DRs were described which were unknown before, at least 13 are likely to be detected due to the new onsite retinopathy screening procedure. This number is rather underestimated due to the fact that underlying progression rate derives from DN and DP which have been shown to progress faster than DR does [33].

The prevalence of DR presented here is in line with current literature [34–37]. In a recent meta-analysis of population-based studies worldwide, an overall estimate for DR of 35% was reported [34]. However, this analysis comprises also rather rural areas and countries with lower developed health care systems, which might explain the slightly higher prevalence compared to our results. In contrast, a lower prevalence of 10.6% for DR has been previously reported from German data, which could be explained by a shorter diabetes duration in that study population compared to the study presented [35].

One might argue that for diabetic retinopathy screening an ophthalmologist is essential and digital fundus imaging alone might not be sufficient. However, recent studies comparing nonophthalmologists with ophthalmologists for the diagnosis of DR have shown similar accuracy in the detection of changes of the fundus even with paramedical staff performing the screening procedure [25, 38–40]. This was also the case in the study presented here. Evaluation of the fundus photography by a reference ophthalmologist revealed that no change of diagnoses of DR categories was necessary (data not shown). Furthermore, several studies have evaluated the sensitivity and specificity of digital fundus screening [20, 41], showing that instant fundus screening by trained nonophthalmologists was accurate and safe in the detection of DR [25, 42]. However, since the number of detected DRs in this study is clearly higher than preexistent DRs, it seems unlikely that a remarkable proportion of patients with DR might have been missed in the study although this cannot be fully excluded, since stereoscopic colour fundus photographs as gold standard were not used in this evaluation.

When analysing factors contributing to the detection of new DR, younger patients' age and shorter diabetes duration were associated with nonattendance of regular screenings and with higher incidence of DR. This is in line with previously reported data showing lower screening attendance rates in younger patients with diabetes [11, 43]. Therefore, we speculate that especially younger patients might benefit from an onsite complications screening including DR since this is time saving and convenient for quickly updating the state of disease and its microvascular complications [44].

However, there are also some limitations to this study. First, since only outpatients were included at a university hospital clinic, participants might not reflect the general diabetes population, which might differ in age and diabetes duration. Therefore, the rate of missed DR might differ, making generalization of the results difficult.

Additionally the study was performed in an area with a higher density of ophthalmologists than the mean of the country [45]. One can speculate that the results observed here might even be more pronounced when density of ophthalmologists is lower, potentially making this screening procedure even more important in other regions, especially in rural areas.

A further issue is high rate of arterial hypertension in the analysed population. The hypertension rates were about 90% of the total patients. As hypertension is a major systemic risk factor for diabetic retinopathy a certain influence on our results cannot be fully excluded and the results might not be so prominent in populations with a lower rate of hypertension. However, rates for arterial hypertension found in our study are comparable to those found in other studies from different regions of the world with hypertension rates up to 95% in patients with diabetes [34, 46, 47].

Furthermore, as data on screening intervals was collected via patient's self-reports and medical data records, a bias of social desirability is possible.

Little is known concerning the effect of onsite screening with a nonmydriatic fundus camera in specialized diabetes outpatient clinics on early diagnosis of DR. The data presented imply that on the one hand this might result in an earlier diagnosis of complications. On the other hand one might speculate that it vice versa might also affect treatment quality and therapy goals, which have been shown to lower the progression of disease [48, 49]. In this study, mean HbA1c levels of patients with newly detected retinopathy were off guideline-implemented target with 8.0% (64 mmol/mol) in patients with diabetes type 1 and 8.4% (68 mmol/mol) in patients with diabetes type 2 [50]. Therefore, further studies have to evaluate the effect of onsite screening on subsequent quality of diabetes treatment when state of complications is immediately known.

In conclusion, the results of this study suggest that onsite screening for diabetic retinopathy with a nonmydriatic digital fundus camera in a diabetes outpatient clinic detects missed diabetic retinopathies in a higher degree than by progression of the disease alone. Due to the epidemic burden of diabetes early identification of patients at risk might help to save time and resources and channel patients with a strong need for specialized eye care especially in younger patients who might profit from time saving diabetes care.

## Figures and Tables

**Table 1:** Baseline parameters of patients with type 1 and type 2 diabetes.

Characteristics	DM type 1 *n* = 112	DM type 2 *n* = 390
Diabetes duration, y	28 ± 15	14 ± 10
≤10 years, *n* (%)	12 (10.7%)	166 (42.6%)
11–19 years, *n* (%)	24 (21.4%)	133 (34.1%)
≥20 years, *n* (%)	76 (67.9%)	91 (23.3%)
Gender, m/w (% of total)	56/56 (50.0%/50.0%)	196/194 (50.3%/49.7%)
Age, mean (SD), y	52 ± 17	65 ± 12
Age, *n* (%)		
≤60 years	74 (66.1%)	129 (33.1%)
61–64 years	5 (4.5%)	60 (15.4%)
65–69 years	11 (9.8%)	46 (11.8%)
70–74 years	14 (12.5%)	58 (14.9%)
75–79 years	5 (4.5%)	56 (14.4%)
≥80 years	3 (2.7%)	41 (10.5%)
BMI, kg/m²	25.7 ± 3.9	33.0 ± 8.2
Hypertension, *n* (%)	59 (52.7%)	347 (89.0%)
Systolic blood pressure, mmHg	135 ± 15	138 ± 19
Diastolic blood pressure, mmHg	81 ± 10	81 ± 12
Nicotine use, *n* (%)	8 (7.1%)	41 (10.5%)
History of cerebrovascular disease, *n* (%)	4 (3.6%)	43 (11.0%)
History of cardiovascular disease, *n* (%)	11 (9.8%)	114 (29.2%)
HbA1c (%)	7.4 ± 1.1	7.3 ± 1.5
Triglyceride level (mg/dL)	94 ± 55	213 ± 217
Total cholesterol level (mg/dL)	180 ± 35	181 ± 40
LDL cholesterol level (mg/dL)	91 ± 26	94 ± 32
HDL cholesterol level (mg/dL)	70 ± 22	48 ± 14
Antidiabetic treatment, *n* (%)		
Lifestyle	0 (0%)	34 (8.7%)
Oral agents	0 (0%)	159 (40.8%)
Insulin treatment	112 (100%)	197 (50.5%)

**Table 2:** Baseline parameters of prevalence and severity of retinopathy in persons with type 1 diabetes (*n* = 112).

Characteristics	DM 1 (*n* = 112)	DR preexisting *n* = 34 (30.4%)	DR new *n* = 6 (5.4%)	*p*_1_	DR all *n* = 40 (35.7%)	DR absent *n* = 72 (64.3%)	*p*_2_	*p*_3_
Gender, *n* (%)								
Male	56 (50.0%)	20 (58.8%)	4 (66.7%)	0.72^*∗*^	24 (60.0%)	32 (44.4%)	0.12^*∗*^	0.29^*∗*^
Female	56 (50.0%)	14 (41.2%)	2 (33.3%)		16 (40.0%)	40 (55.6%)		
Age, y	52 ± 17	64 ± 11	51 ± 11	0.01^∧^	62 ± 12	47 ± 17	**<**0.001^∧^	0.58^∧^
Age, *n* (%)								
≤60 years	74 (66.1%)	13 (38.2%)	5 (83.3%)	0.04^*∗*^	18 (45.0%)	56 (77.8%)	**<**0.001^*∗*^	0.75^*∗*^
≥80 years	3 (2.7%)	3 (8.8%)	0	0.45^*∗*^	3 (7.5%)	0	0.02^*∗*^	1.00
Diabetes duration, y	28 ± 15	40 ± 12	29 ± 8	0.05^∧^	38 ± 12	23 ± 13	**<**0.001^∧^	0.26^∧^
≤10 years, *n* (%)	12 (10.7%)	0	0	1.00	0	12 (16.7%)	0.01^*∗*^	0.28^*∗*^
11–19 years, *n* (%)	24 (21.4%)	2 (5.9%)	1 (16.7%)	0.36^*∗*^	3 (7.5%)	21 (29.2%)	0.01^*∗*^	0.51^*∗*^
≥20 years, *n* (%)	76 (67.9%)	32 (94.1%)	5 (83.3%)	0.36^*∗*^	37 (92.5%)	39 (54.2%)	**<**0.001^*∗*^	0.17^*∗*^
Median time to last eye screening (months)	6.5 ± 7.6	4.2 ± 4.0	7.0 ± 3.1	0.12^∧^	4.7 ± 4.0	7.6 ± 8.9	0.05^∧^	0.87^∧^
Hypertension, *n* (%)	59 (52.7%)	30 (88.2%)	4 (66.7%)	0.17^*∗*^	34 (85.0%)	25 (34.7%)	**<**0.001^*∗*^	0.12^*∗*^
Systolic blood pressure, mmHg	135 ± 15	137 ± 16	146 ± 13	0.21^∧^	139 ± 16	133 ± 14	0.05^∧^	0.03^∧^
Diastolic blood pressure, mmHg	81 ± 10	78 ± 10	91 ± 8	0.005^∧^	80 ± 11	81 ± 10	0.41^∧^	0.04^∧^
Statin therapy	51 (45.5%)	25 (73.5%)	2 (33.3%)	0.05^*∗*^	27 (67.5%)	24 (33.3%)	0.001^*∗*^	1.00^*∗*^
Biochemical characteristics								
Total cholesterol level (mg/dL)	180 ± 35	169 ± 33	212 ± 42	0.01^∧^	175 ± 37	182 ± 33	0.30^∧^	0.04^∧^
HbA1c (%)	7.4 ± 1.1	7.1 ± 1.2	8.0 ± 1.2	0.12^∧^	7.2 ± 1.2	7.4 ± 1.1	0.38^∧^	0.91^∧^

DR = diabetic retinopathy, HDL = high density lipoprotein, and LDL = low density lipoprotein.

*p*_1_ = *p* value DR preexisting versus DR new.

*p*_2_ = *p* value DR all versus DR absent.

*p*_3_ = *p* value DR new versus DR absent.

^*∗*^Chi-square test (*χ* ^2^-test), ^∧^ *t*-test.

**Table 3:** Baseline parameters of prevalence and severity of retinopathy in persons with type 2 diabetes (*n* = 390).

Characteristics	DM 2 (*n* = 390)	DR preexisting *n* = 60 (15.4%)	DR new *n* = 26 (6.7%)	*p*_1_	DR all *n* = 86 (22.1%)	DR absent *n* = 304 (77.9%)	*p*_2_	*p*_3_
Gender, *n* (%)								
Male	196 (50.3%)	29 (48.3%)	14 (53.8%)	0.64^*∗*^	43 (50%)	153 (50.3%)	0.957^*∗*^	0.731^*∗*^
Female	194 (49.7%)	31 (51.7%)	12 (46.2%)		43 (50%)	151 (49.7%)		
Age, y	65 ± 12	71 ± 9	66 ± 11	0.07^∧^	69 ± 9	64 ± 12	**<**0.001^∧^	0.285^∧^
Age, *n* (%)								
≤60 years	129 (33.1%)	9 (15.0%)	10 (38.5%)	0.02^*∗*^	19 (12.8%)	110 (74.3%)	0.01^*∗*^	0.82^*∗*^
≥80 years	41 (10.5%)	11 (18.3%)	3 (11.5%)	0.43^*∗*^	14 (25.5%)	27 (49.1%)	0.05^*∗*^	0.65^*∗*^
Diabetes duration, y	14 ± 10	24 ± 10	18 ± 11	0.01^∧^	22 ± 10	11 ± 8	**<**0.001^∧^	**<**0.001^∧^
≤10 years, *n* (%)	166 (42.6%)	4 (6.7%)	5 (19.2%)	0.08^*∗*^	9 (10.5%)	157 (51.6%)	**<**0.001^*∗*^	0.002^*∗*^
11–19 years, *n* (%)	133 (34.1%)	16 (26.7%)	13 (50.0%)	0.04^*∗*^	29 (33.7%)	104 (34.2%)	0.93^*∗*^	0.11^*∗*^
≥20 years, *n* (%)	91 (23.3%)	40 (66.7%)	8 (30.8%)	0.002^*∗*^	48 (55.8%)	43 (14.1%)	**<**0.001^*∗*^	0.02^*∗*^
Median time to last eye screening (months)	9 ± 13	5 ± 12	11 ± 11	0.05^∧^	7 ± 12	10 ± 13	0.04^∧^	0.86^∧^
Hypertension, *n* (%)	347 (89.0%)	59 (98.3%)	24 (92.3%)	0.16^*∗*^	83 (96.5%)	264 (86.8%)	0.01^*∗*^	0.42^*∗*^
Antidiabetic treatment, *n* (%)								
Lifestyle	34 (8.7%)	1 (1.7%)	2 (7.7%)	0.16^*∗*^	3 (3.5%)	31 (10.2%)	0.05^*∗*^	0.68^*∗*^
Oral agents	261 (66.9%)	28 (46.7%)	19 (73.1%)	0.02^*∗*^	47 (54.7%)	214 (70.4%)	0.006^*∗*^	0.78^*∗*^
Insulin treatment	197 (50.5%)	52 (86.7%)	19 (73.1%)	0.13^*∗*^	71 (82.6%)	126 (41.4%)	**<**0.001^*∗*^	0.002^*∗*^
Statin therapy	237 (60.8%)	41 (68.3%)	19 (73.1%)	0.66^*∗*^	60 (69.8%)	177 (58.2%)	0.05^*∗*^	0.14
HbA1c (%)	7.3 ± 1.5	7.8 ± 1.5	8.4 ± 2.0	0.18^∧^	8.0 ± 1.7	7.1 ± 1.5	**<**0.001^∧^	0.003^∧^

DR = diabetic retinopathy.

HDL = high density lipoprotein, LDL = low density lipoprotein.

*p*_1_ = *p* value DR preexisting versus DR new.

*p*_2_ = *p* value DR all versus DR absent.

*p*_3_ = *p* value DR new versus DR absent.

^*∗*^Chi-square test (*χ* ^2^-test).

^∧^ *t*-test.

**Table 4:** Progression rate of DR, DN, and DP for all patients with diabetes was calculated as ratio of new detected complications to preexistent complications.

	New detected (*n*)	Preexistent (*n*)	Progression rate (%)
Diabetic retinopathy	32	94	34.0
Diabetic neuropathy	53	266	19.9
Diabetic nephropathy	29	134	21.6

**Table 5:** Odds ratio (95% CI) of failure to attend eye screening within one year.

Characteristics	All (*n* = 67)	*p*_1_	Type 2 (*n* = 60)	*p*_2_
Gender				
Male	0.91 (0.54–1.52)	0.72	0.935 (0.54–1.62)	0.81
Female				
Age				
≤60	2.00 (1.19–3.36)	**0.01**	2.33 (1.33–4.07)	**0.002**
61–64	1.57 (0.79–3.13)	0.19	1.47 (0.73–3.00)	0.28
65–69	0.46 (0.16–1.31)	0.14	0.49 (0.17–1.42)	0.18
70–74	0.08 (0.01–0.57)	**0.001**	0.08 (0.01–0.60)	0.002
75–79	1.14 (0.54–2.44)	0.73	1.06 (0.49–2.30)	0.88
≥80	0.63 (0.22–1.81)	0.39	0.57 (0.19–1.65)	0.29
Diabetes duration				
≤10 years	4.30 (2.50–7.40)	**<0.001**	3.54 (1.97–6.37)	**<0.001**
11–19 years	0.65 (0.36–1.19)	0.16	0.48 (0.25–0.93)	0.027
≥20 years	0.20 (0.09–0.45)	**<0.001**	0.32 (0.13–0.77)	**0.008**
Diabetic retinopathy	0.42 (0.20–0.88)	**0.02**	0.50 (0.23–1.09)	0.08
Preexistent	0.25 (0.08–0.83)	**0.02**	0.25 (0.08–0.83)	**0.02**
New diagnosed	1.22 (0.45–3.28)	0.70	1.34 (0.48–3.70)	0.75

## Abbreviations

DN: Diabetic nephropathy
DP: Diabetic polyneuropathy
DR: Diabetic retinopathy.
